# ACD-Net: An Abnormal Crew Detection Network for Complex Ship Scenarios

**DOI:** 10.3390/s24227288

**Published:** 2024-11-14

**Authors:** Zhengbao Li, Heng Zhang, Ding Gao, Zewei Wu, Zheng Zhang, Libin Du

**Affiliations:** 1 College of Ocean Science and Engineering, Shandong University of Science and Technology, No. 579 Qianwan Port Road, Huangdao District, Qingdao 266590, China; lizhengbao@sdust.edu.cn (Z.L.); 202282190005@sdust.edu.cn (H.Z.); gaoding888@outlook.com (D.G.); zeweiwu@sdust.edu.cn (Z.W.); 2 Yellow Sea Fisheries Research Institute, Chinese Academy of Fishery Sciences, 106 Nanjing Road, Qingdao 266071, China; zhangzheng@ysfri.ac.cn

**Keywords:** abnormal behavior detection, identity recognition, YOLOv5s, facial images, face quality assessment

## Abstract

Abnormal behavior of crew members is an important cause of frequent ship safety accidents. The existing abnormal crew recognition algorithms are affected by complex ship environments and have low performance in real and open shipborne environments. This paper proposes an abnormal crew detection network for complex ship scenarios (ACD-Net), which uses a two-stage algorithm to detect and identify abnormal crew members in real-time. An improved YOLOv5s model based on a transformer and CBAM mechanism (YOLO-TRCA) is proposed with a C3-TransformerBlock module to enhance the feature extraction ability of crew members in complex scenes. The CBAM attention mechanism is introduced to reduce the interference of background features and improve the accuracy of real-time detection of crew abnormal behavior. The crew identification algorithm (CFA) tracks and detects abnormal crew members’ faces in real-time in an open environment (CenterFace), continuously conducts face quality assessment (Filter), and selects high-quality facial images for identity recognition (ArcFace). The CFA effectively reduces system computational overhead and improves the success rate of identity recognition. Experimental results indicate that ACD-Net achieves 92.3% accuracy in detecting abnormal behavior and a 69.6% matching rate for identity recognition, with a processing time of under 39.5 ms per frame at a 1080P resolution.

## 1. Introduction

Maritime shipping activities have become increasingly prosperous, accompanied by frequent maritime accidents during the development of the marine economy. The report “Preliminary Overview of Maritime Injuries and Accidents 2014–2020” by the European Maritime Safety Agency (EMSA) [1] states that a total of 22,532 maritime accidents occurred over a period of 7 years. In total, 51% of these accidents resulted in serious casualties, including 1650 deaths and 20,763 disabilities. The report highlights that human error [2] is the primary cause of maritime casualties, with accidents due to abnormal crew behavior accounting for 79% of human error incidents. Identifying abnormal behavior and crew members can reduce human errors caused by crew abnormalities, lower the likelihood of accidents, and provide critical support for accident warning and process analysis.

In recent years, many algorithms have been proposed in the fields of abnormal behavior detection and identity recognition. These algorithms can be divided into three categories based on the different collection devices. Personnel wearing portable smart sensing devices (such as health monitors and WiFi devices) have certain advantages in detecting abnormal behavior and identifying individuals in open areas through the collected physiological signals (heart rate, gait, etc.) [3,4,5,6]. The complex onboard environment (such as humidity and vibration, etc.) can lead to sensor failures, and the limitations of equipment lifespan and network connectivity can affect the timeliness and accuracy of data. Therefore, this type of method is limited in the application of onboard personnel identification.

Video surveillance devices widely employ computer vision technology for anomaly detection and identity recognition applications. Video-based detection methods can extract features in the temporal dimension [7,8,9,10]. Although these methods demonstrate good detection accuracy, they require high computational power, making them less suitable for onboard applications. In contrast, image-based detection methods [11,12,13] maintain high accuracy while having lower computational demands, making them more appropriate for onboard monitoring.

Biometric devices, such as fingerprint scanners and iris scanners, are used for identity verification through biological features [14,15,16,17]. Although these biometric identification methods offer high accuracy, they require active cooperation from individuals and are suitable for scenarios with high security requirements. Additionally, the complex maritime environment, including factors, such as lighting variations and humidity, may affect the accuracy of iris recognition.

**Figure 1:**
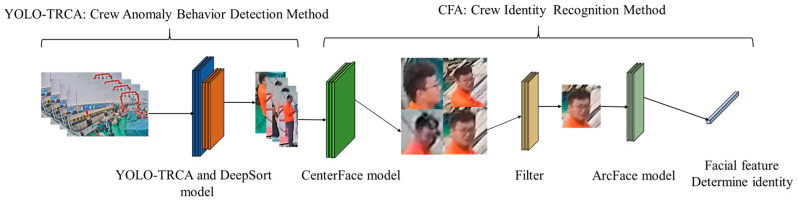
ACD-Net: Abnormal crew detection network.

This paper proposes an abnormal crew detection network for complex ship scenarios (ACD-Net), which is used to detect abnormal behavior and for identity recognition of crew members. The recognition process of ACD-Net is shown in Figure 1. The improved YOLOv5s model based on a transformer and CBAM mechanism (YOLO-TRCA) is used to detect abnormal behavior of crew members and track them through Deepsort. The crew identity recognition algorithm (CFA) comprises face detection (CenterFace), face quality assessment (Filter), and face recognition (ArcFace) to identify abnormal crew identities. The innovations of the paper are as follows: (1) A method called YOLO-TRCA has been proposed for real-time detection of abnormal crew behavior. YOLO-TRCA adopted a TransformerBlock self-attention mechanism [18] improved feature extraction network, introduced a channel-spatial attention mechanism (CBAM) [19], and combined with a CIoU loss function to realize abnormal behavior recognition of crew. (2) We innovatively incorporate a fast face quality assessment algorithm called Filter into our identity recognition method (CFA). Filter can effectively avoid the interference of low-quality faces in the pre-screening stage, reduce computational costs, and improve the recognition accuracy of the method.

## 2. Related Work

Traditional image-based anomaly behavior detection methods are categorized into manual feature detection, dynamic Bayesian networks (DBN) [20,21], clustering models [22], and sparse representation methods [23]. These algorithms perform well in simple scenarios but struggle in complex application tasks. Deep learning-based anomaly detection models are widely used to identify targets exhibiting abnormal behavior in images. Region-based convolutional neural networks (Faster-RCNN) [24] utilize a two-stage structure, which requires a large amount of computation and is unsuitable for real-time processing scenarios. The single shot multibox detector (SSD) [25] experiences a high false positive rate in complex backgrounds. End-to-end detection models in the YOLO series [26,27,28,29,30] achieve relatively high accuracy and speed, making them suitable for real-time applications. RetinaNet [31] has high accuracy in recognizing small objects. CenterNet [32] predicts targets through center point detection, suitable for dense scenes, but has a slower inference speed than YOLO and SSD. The confirmation of crew identity is an important part of identifying abnormal behavior among crew members. Image-based identity recognition algorithms primarily identify individuals through facial features in images. Deep learning-based face detection mainly includes cascade CNN face detection [33], which filters face regions in multiple stages but is ineffective for small faces. MTCNN [34] performs well in detecting faces of different scales but is sensitive to lighting and expression changes, affecting detection performance. Faster R-CNN face detection [35] can handle faces in complex scenes but has poor real-time performance. CenterFace [36] can manage densely populated faces with good real-time capabilities but is easily influenced by pose and lighting changes. Face recognition primarily involves facial feature extraction models. For instance, FaceNet [37] has high detection accuracy but is affected by ambient light. The VGG Face [38] network is deeper, resulting in slower inference speeds. ArcFace [39] exhibits strong robustness to lighting variations, pose changes, and occlusions, making it suitable for various application scenarios.

In recent years, researchers have conducted several studies on crew behavior detection and identity recognition based on image data. Wawrzyniak et al. developed a vessel monitoring system that uses background subtraction and feature matching techniques to detect and track vessels on the sea surface in order to address interference from maritime lighting and weather conditions [40]. This algorithm provides the idea for crew behavior detection and identity recognition in onboard environments with varying lighting conditions. Kim et al. designed a deep learning algorithm to identify vessels and crew, as well as to detect abnormal overboard behavior of crew members. However, this algorithm did not consider the impact of environmental factors, image jitter, and obstructions on overboard incidents [11]. Chen Xinqiang et al. employed an asynchronous interactive aggregation network to detect abnormal behavior of personnel in port operation areas, taking into account complex port scenarios with low light and crew obstructions [12]. This network can accurately identify typical abnormal behaviors in port working environments, but its performance declines when crew members are significantly or completely obscured. Rizk et al. developed an enhanced YOLOv4 model for the real-time detection of individuals floating in the ocean after falling overboard [13]. This algorithm achieved good results by integrating a dedicated AI application onto a low-power hardware platform for personnel detection, which is applicable to the field of maritime search and rescue and provides valuable insights for detecting abnormal personnel onboard. Park et al. introduced a passenger ship safety management system that combines face recognition and gesture recognition to identify passengers and crew, facilitating efficient management of passenger and vessel safety [16]. Luan et al. employed face recognition technology for crew identification, effectively addressing the issue of counterfeit registration documents. However, this method is not applicable to ship monitoring videos [41]. Jayavadivel et al. proposed a facial recognition method based on low-resolution images for face and iris biometric identification [17]. This method is used for the automatic recognition and verification of individuals with frontal faces and irises captured by the camera, under certain constraints.

In summary, there are still several limitations in the research on theories and methods for detecting abnormal crew behavior and facial recognition. (1) The algorithms for detecting abnormal behavior and identifying crew members do not adequately account for lighting conditions and image shake. The performance of abnormal behavior detection diminishes in complex monitoring environments where crew members are significantly obscured. Crew facial recognition is conducted in highly constrained scenarios, making it unsuitable for unrestrained maritime monitoring environments. (2) Limited equipment performance on ships makes it difficult to deploy large detection models for real-time crew behavior monitoring and face recognition, while smaller detection models generally produce suboptimal results on ships. To address these issues, we propose a combined abnormal crew detection network that can identify both abnormal behaviors and the identities of the affected crew members simultaneously.

## 3. Methodology

### 3.1. Crew Anomaly Behavior Detection

#### 3.1.1. Comparison of Related Detection Models

Accuracy and speed are key performance indicators for evaluating algorithms in the field of object detection. We have selected several algorithms with good performance, such as YOLOv5, Faster R-CNN, SSD, RetinaNet, and YOLOv4. The performance evaluation results of these algorithms on different datasets are shown in Table 1. The dataset includes multiple datasets, such as COCO, Pascal VOC, and ImageNet, covering a wide range of target categories and scenarios. From Table 1, it can be seen that YOLOv5 has the highest accuracy on the ImageNet dataset, while Faster R-CNN slightly leads on the COCO dataset, and YOLOv4 performs better on the Pascal VOC dataset. YOLOv5 is similar to other algorithms in terms of accuracy, and even has a slight advantage.

Real-time inference speed is essential for the practical application of object detection algorithms. Table 2 presents the inference speed results of YOLOv5 and other algorithms on GPU. It is evident that YOLOv5 achieves the fastest inference speed on the NVIDIA GeForce GTX 1080 Ti. A comprehensive analysis of Table 1 and Table 2 indicates that YOLOv5 achieves a good balance between speed and accuracy, making it suitable for the application scenario of shipborne abnormal behavior detection.

#### 3.1.2. Analysis of Crew Abnormal Behaviors Detection Issues Based on YOLOv5

We analyzed the operating procedures and on-site operations of the crew and determined the abnormal behaviors that need to be identified in this article based on the frequency and degree of harm of the behavior, including not wearing work clothes, not wearing life jackets, exposing upper bodies, smoking, etc. YOLOv5s was selected to detect abnormal crew behavior in images, taking into account the computing power and real-time requirements of onboard equipment. It is a single-stage object detection algorithm that maintains high accuracy while providing real-time performance, consisting of four parts: the input, backbone, neck, and head.

**Figure 2:**
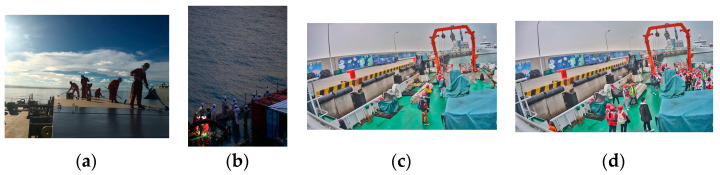
Four types of images with distinct features: (**a**) image with uneven lighting and significant brightness variations; (**b**) image with local overexposure, underexposure, or blurring; (**c**) image with a cluttered background and a small proportion of crew images; (**d**) image with severe occlusions and overlaps between crew and equipment.

We selected four types of images with distinct features based on the previously analyzed shipboard image characteristics and employed YOLOv5s for recognition. Performance analysis was conducted on the prediction results and Grad-CAM feature visualization. Figure 2a shows an image with uneven lighting and significant brightness variations. Figure 2b depicts an image with local overexposure, underexposure, or blurring. Figure 2c represents an image with a cluttered background and a small proportion of crew images. Figure 2d illustrates an image with severe occlusions and overlaps between crew and equipment. The recognition accuracies of the YOLOv5s model for these images (Figure 2a–d) were 66.7%, 54.5%, 66.7%, and 39.3%, respectively. The accuracy of detecting crew abnormal behavior was 56.8%, which is low, and which struggles to meet practical needs.

**Figure 3:**
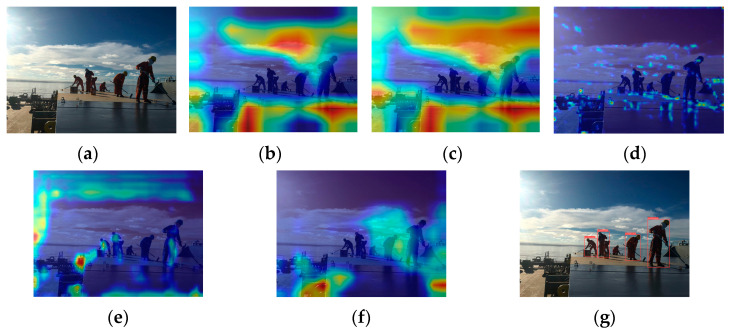
YOLOv5s feature visualization and recognition effect diagram: (**a**) original image; (**b**) the C3 model before SPPF; (**c**) SPPF; (**d**) input 1 of the neck (PAN); (**e**) input 2 of the neck (PAN); (**f**) input 3 of the neck (PAN); (**g**) YOLOv5s detection diagram.

There are a total of 6 recognition targets (Figure 3). YOLOv5s identified 4 of these targets with an accuracy of 66.7%. The crew members at the farthest distance and those obscured were not detected, as shown in Figure 3g. Analysis of the original image in Figure 3a reveals that the overall lighting distribution in the image is uneven. The left side of the image is brightly lit, while the crew member images have low contrast and are relatively dark. This similarity in color characteristics between the crew members and other black objects on the deck increases the difficulty of feature extraction and recognition. The proportion of the farthest squatting crew member and the obstructed crew member in the entire image is relatively small, making it challenging to extract and distinguish crew behavior features. Figure 3b–f shows the feature visualization results of Figure 3a. Figure 3c is a feature visualization of the last layer of the backbone network (SPPF). It can be analyzed from the figure that SPFF focuses on the sky and deck areas and pays less attention to the crew working on board during the feature extraction process. This will result in insufficient feature extraction capabilities for the targets. Comparing the feature visualization results of SPPF and the C3 model before SPPF (Figure 3b,c) shows that the regions of interest remain largely unchanged. This indicates that the features extracted by the C3 model before SPPF influence the final output features of SPPF. In the feature fusion process, input 1 of the neck (PAN) (Figure 3d) shows little attention to the crew member areas. At this point, there were very few crew features left, only background features. Input 2 of the neck (PAN) (Figure 3e) focuses on 3 crew members, and input 3 of the neck (PAN) (Figure 3f) focuses on 2 crew members, but neither input identifies the crew member crouched at a distance. This indicates that the crew member’s features disappeared during the feature fusion stage, resulting in their failure to be recognized.

**Figure 4:**
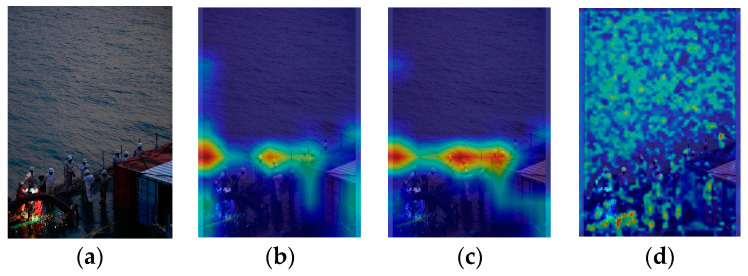

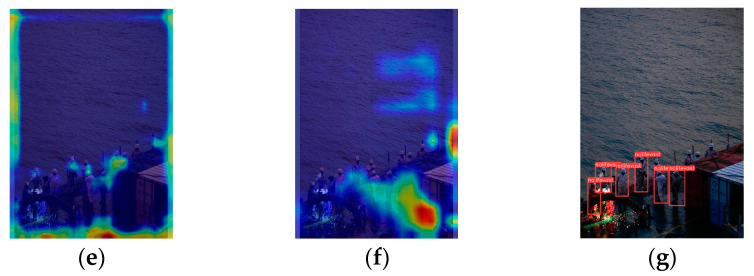
YOLOv5s feature visualization and recognition effect diagram: (**a**) original image; (**b**) the C3 model before SPPF; (**c**) SPPF; (**d**) input 1 of the neck (PAN); (**e**) input 2 of the neck (PAN); (**f**) input 3 of the neck (PAN); (**g**) YOLOv5s detection diagram.

There are a total of 11 recognition targets in Figure 4, and YOLOv5s identified 6 of these targets with an accuracy of 54.5%. Crew members in overexposed areas and those obscured by containers were not detected (Figure 4g). Figure 4a was taken during the evening, and insufficient lighting caused local underexposure in some shadowed areas on the ship, leading to the loss of crew detail features. Some areas of the image are overexposed, making it difficult to extract crew features. Certain crew members are obscured by containers, with only their heads visible. This makes feature extraction extremely challenging due to their small portion of the image. The feature visualization results in Figure 4b–f illustrate the issues with feature extraction and fusion. During the feature extraction process, the feature visualization of the SPPF focuses primarily on the six central crew members and parts of the environment on the left (Figure 4c), without capturing features of all crew members. Comparing the feature visualization results of SPPF and the C3 model before SPPF (Figure 4b,c) shows that the areas of interest for the C3 model before SPPF are largely consistent with those of SPPF. In the process of feature fusion, input 1 of the neck (PAN) (Figure 4d) does not emphasize any particular regions of the image. It indicates that the crew member features are not prominent during feature fusion. Input 2 of the neck (PAN) (Figure 4e) identifies 6 crew members, and input 3 of the neck (PAN) (Figure 4f) identifies 4 crew members. However, none of the inputs addresses the crew members obscured by containers or those in overexposed areas, leading to lower attention to these targets during recognition.

**Figure 5:**
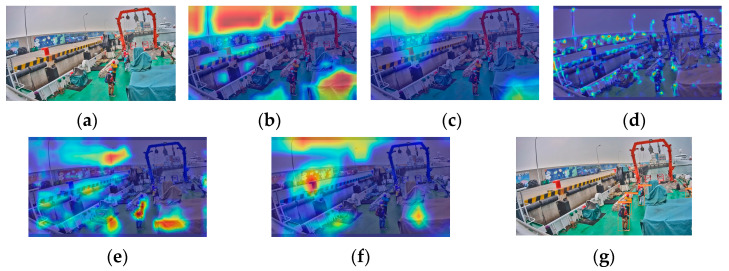
YOLOv5s feature visualization and recognition effect diagram: (**a**) original image; (**b**) the C3 model before SPPF; (**c**) SPPF; (**d**) input 1 of the Neck (PAN); (**e**) input 2 of the Neck (PAN); (**f**) input 3 of the Neck (PAN); (**g**) YOLOv5s detection diagram.

YOLOv5s detected 4 out of 6 targets in Figure 5, with an accuracy of 66.7%. The two overlapping crew members in the center shown in Figure 5g were not detected. We can see from Figure 5a that the background on the ship is cluttered. The crew member on the right is partially obscured by equipment, which makes it difficult to extract complete features of this crew member. The clothing of the obscured crew member is similar in color to the deck, making it challenging to extract the crew member’s color features. The overlap between crew members makes it hard to distinguish behavioral features. Figure 5b–f shows the feature visualization results of Figure 5a during the feature extraction and fusion stages. The feature visualization of the SPPF (Figure 5c) focuses mainly on the background area rather than the working crew members on the ship during feature extraction. This indicates that background features are overemphasized, weakening the extraction of target features. Comparing the feature visualization results of SPPF and the C3 model before SPPF (Figure 5b,c), it is observed that the specific areas of focus are roughly the same. During the feature fusion process, input 1 of the neck (PAN) (Figure 5d) identifies 4 crew members, but only the frontmost crew member is noted among the 3 overlapping ones. Input 2 of the neck (PAN) (Figure 5e) identifies 2 crew members, with the obscured crew member and the 3 overlapping crew members in the middle not being addressed. Input 3 of the neck (PAN) (Figure 5f) does not focus on the crew member areas, introducing excessive background feature interference. This results in reduced attention to targets in subsequent recognition.

**Figure 6:**
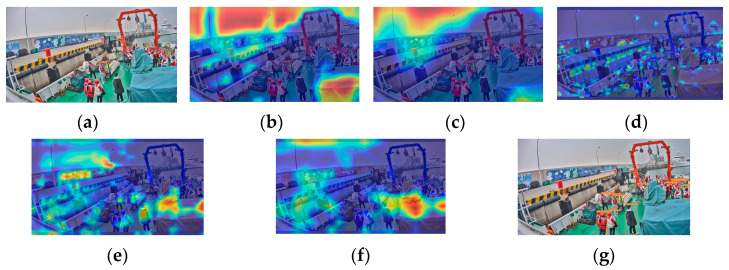
YOLOv5s feature visualization and recognition effect diagram: (**a**) original image; (**b**) the C3 model before SPPF; (**c**) SPPF; (**d**) input 1 of the neck (PAN); (**e**) input 2 of the neck (PAN); (**f**) input 3 of the neck (PAN); (**g**) YOLOv5s detection diagram.

YOLOv5s achieved the lowest recognition accuracy of 39.3% in Figure 6, identifying 11 out of 28 recognition targets. Most crew members obscured by equipment and those with severe overlapping are not detected in the recognition image (Figure 6g). Analysis of the original image (Figure 6a) shows that the complex background onboard and the obscured crew members make feature extraction difficult. The crew members are densely concentrated in a small area of the image, with overlapping members being predominant, making it challenging to extract all crew member features. From the feature visualization effects of Figure 6b–f, it can be seen that there are issues in the processes of feature extraction and fusion. During the feature extraction process, the SPPF focuses on the shore environment and some container areas rather than the working crew on the ship (Figure 6c). This results in significant interference from background features. Comparing the feature visualization effects of SPPF and the C3 model before SPPF (Figure 6b,c), the regions of focus are roughly similar. It indicates that the features extracted by the C3 model before SPPF influence the final output features of SPPF. During the feature fusion process, input 1 of the neck (PAN) (Figure 6d) identifies 11 crew members, but the attention to the concentrated crew area is low. Furthermore, most overlapping crew members in this region are not addressed. Input 2 of the neck (PAN) (Figure 6e) identifies 5 crew members, and input 3 of the neck (PAN) (Figure 6f) identifies 3 crew members. The crew concentration areas are not attended to, with excessive attention given to the background. This leads to severe interference from background features, resulting in a significantly lower recognition rate for the targets in the crew concentration area.

We comprehensively analyzed the four types of images with significant features mentioned above and found that applying YOLOv5s to crew abnormal behavior detection has the following common defects. (1) The SPPF focuses more on the environment rather than the crew area, and the final output features of SPPF are mainly influenced by the features extracted by the C3 model before SPPF. The C3 model before SPPF has insufficient capability for extracting crew features in the complex noise environment on the ship. (2) The attention to the crew area is relatively low during feature fusion, with excessive focus on the background area. Background features cause severe interference with crew features. The above analysis indicates that the original YOLOv5s fails to accurately extract and distinguish crew behavior features. This is the direct cause of its low detection accuracy for crew abnormal behavior.

#### 3.1.3. Feature Extraction Network Based on C3-TransformerBlock

**Figure 7:**
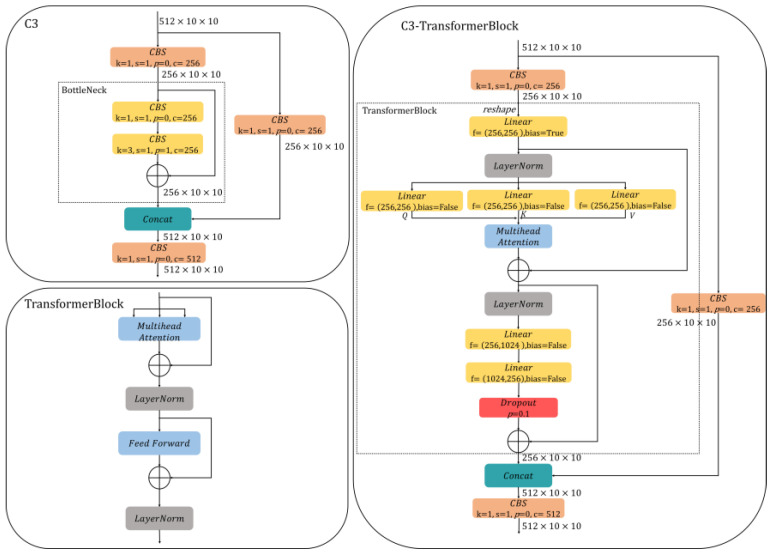
The structures of C3 module, TransformerBlock, and the C3-TransformerBlock module.

The onboard environment is complex, with high variability in lighting conditions leading to local overexposure and underexposure in images. Global contextual information can help the model understand changes in lighting during feature extraction. For example, when a certain part of the image is overexposed, the model can rely on features from the surrounding areas to determine the true color and shape of crew members, thereby reducing the impact of lighting variations on feature extraction. Additionally, blurriness caused by image jitter makes local features unstable. Integrating global contextual information can provide extra spatial data, enabling the model to recognize important crew features even when local information is unclear. YOLOv5 employs the CSPDarknet53 network for feature extraction, focusing on extracting local features, with a limited ability to integrate global contextual information. CSPDarknet53 lacks the ability to extract key features of crew members in complex environments with certain issues, such as the high variability of light on the ship. The nonlinear transformation capability of the C3 module in CSPDarknet53 is insufficient. As illustrated in Figure 7, the BottleNeck in the C3 module before SPPF is a residual structure containing only two CBS, and its limited nonlinear transformation ability restricts the crew anomaly behavior detection model’s adaptability to complex scenarios. The C3 model before SPPF in the YOLOv5 backbone network is being considered for improvement to enhance its feature extraction capability for crew members in complex noise environments, such as those involving light variability and image blurring on board ships.

We combine the C3 model before SPPF in the feature extraction network with the TransformerBlock module to form the C3-TransformerBlock module. It can utilize the ability of the transformer to efficiently integrate global contextual information, without being limited by local perception range, to compensate for the insufficient crew feature extraction ability of CSPDarknet53 in complex scenes. The multi-head attention processes multiple different feature subspaces, which helps enhance non-linear transformation capabilities and improves the crew anomaly behavior detection model’s adaptability in complex ship scenes. The C3-TransformerBlock module (Figure 7) replaces the BottleNeck structure in the C3 module with the TransformerBlock module. The input feature map of size 512 × 10 × 10 is fed into two branches. The convolution kernel size of the CBS is 1 × 1 with 256 channels in the main branch. The output of the CBS is a feature map of size 256 × 10 × 10, which is concatenated with the input from the TransformerBlock module. The TransformerBlock within the C3-TransformerBlock module can be divided into three sublayers. The first sublayer is a linear layer that maps the input features to enhance crew features representation capability. The second sublayer applies LayerNorm for layer normalization to accelerate training and improve crew anomaly behavior detection model stability. It then passes through three linear layers for Q, K, and V, with their outputs fed into the multi-head attention to capture relationships among the crew and background features. The third sublayer also begins with LayerNorm, followed by two linear layers f1 and f2. The f1 expands the channel from 256 to 1024 dimensions, and the f2 reduces it back to 256 dimensions. The nonlinear transformation ability of the crew abnormal behavior detection model is improved by first expanding and then compressing the channel. A dropout with a probability of *p* = 0.1 is applied to randomly drop some neurons to prevent overfitting. Both the second and third sublayers are connected via a residual structure. The output feature map from the TransformerBlock is of size 256 × 10 × 10, which is concatenated with the feature map from the other branch, resulting in an output feature map of size 512 × 10 × 10. Finally, a CBS with a convolution kernel size of 1 × 1 is applied to obtain a feature map of size 512 × 10 × 10. This feature map contains rich contextual information and crew features in complex environments.

#### 3.1.4. Feature Fusion Network Based on a CBAM Attention Mechanism

The monitoring background on board is cluttered, the proportion of crew images is small, and there is similarity between crew behavior characteristics. These issues lead to background feature interference or confusion of different crew behavior features during the multi-scale feature fusion stage (neck) of the original YOLOv5s. In the feature multi-scale fusion stage (neck) of the YOLOv5s model, a path aggregation network (PAN) is used to enhance multi-scale feature fusion capability. During this process, background information from high-level feature maps is fused into low-level feature maps. This fusion causes crew features and background features to mix, affecting detection accuracy.

**Figure 8:**
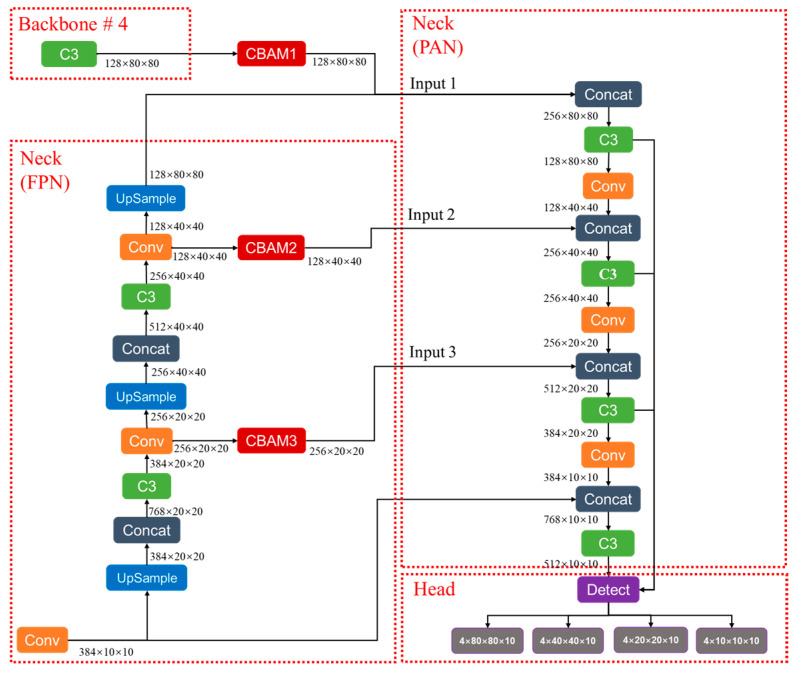
Added CBAM schematic diagram in the feature fusion network.

We have considered adding CBAM (Figure 8) in the input stage of PAN to improve the detection accuracy of abnormal crew members. The input of CBAM1 is the fourth layer of the backbone network, which belongs to shallow features and pays more attention to crew details. The inputs of CBAM2 and CBAM3 are high-level features fused with background information. Properly setting the spatial convolution kernel size can prevent the invalidation of small-scale crew behavior features and reduce computational complexity, while enhancing spatial attention to these features in multi-scale fusion. The spatial convolution kernel size is set to 7 × 7 after experimental comparison. Additionally, the size of the dimensionality reduction coefficient can effectively allocate channel attention and reduce channel computation. The channel dimension reduction coefficient is set to 16 after experimental comparison (Table A1). The CBAM mechanism serially generates attention feature information of crew members in both the channel and spatial dimensions, effectively extracting and preserving critical local features of the image during feature fusion. Channel attention allocates greater weight to differentiate crew features, while spatial attention extracts appearance and behavioral characteristics of the crew at different scales. The combination of these attentions focuses feature on the key information of crew members and suppresses irrelevant feature of background interference. This directs subsequent predictors to concentrate on effective crew regions within both channels and spatial dimensions, thereby enhancing crew anomaly behavior detection model performance.

#### 3.1.5. Loss Function

**Figure 9:**
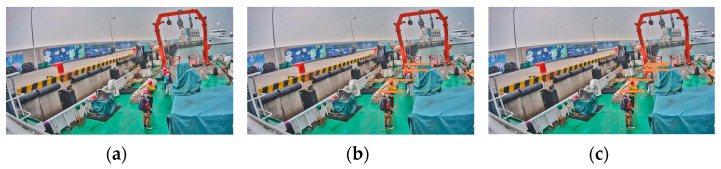
Comparison of loss function effects: (**a**) original image; (**b**) IoU; (**c**) CIoU.

Occlusion by equipment and overlap between crew members are severe due to the unique monitoring environment on the ship. The original YOLOv5 uses the intersection over union (IoU) loss function to select candidate boxes of different crew members by calculating the IoU between boxes. However, it does not consider the distance between the centers of different crew members. This can suppress overlapping candidate boxes for different crew, making it difficult to identify crew members in occluded and overlapping states. The three overlapping crew members should have three close bounding boxes, as shown in Figure 9b. After applying IoU, the bounding box with lower confidence is discarded due to excessive overlap with the higher confidence box, resulting in a detection failure for that crew member.

We consider replacing the IoU loss function with the CIoU loss function, which can effectively reduce the impact of crew occlusion by equipment and overlap between crew members on recognition accuracy. The advantage of using the CIoU loss function is that it not only considers the overlapping area of different detection boxes, but also takes into account the center point distance and aspect ratio of different crew members. This allows candidate boxes with high overlapping areas to be identified as different crew members (Figure 9c).

(1) $$s_{i}=\{\begin{array}{c} s_{i},IoU-R_{CIoU}(M,B_{i})<\varepsilon \\ 0,IoU-R_{CIoU}(M,B_{i})\geq \varepsilon \end{array}$$

In Equation (1), $s_{i}$ represents the predicted confidence score of the ith detection box, ε is the NMS threshold, M is the detection box with the highest confidence score, and Bi is the ith detection box. Taking into account the center distance, ε will be set to 0.45.

#### 3.1.6. Improved Overall Network Structure

**Figure 10:**
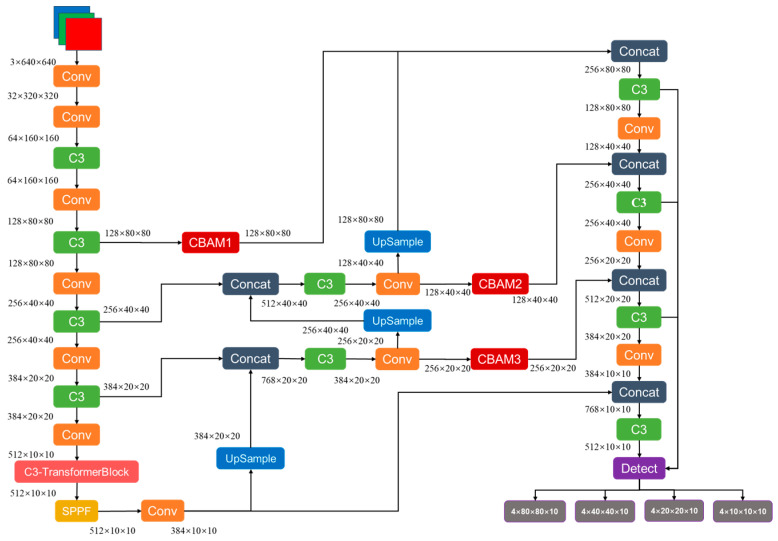
The architecture of YOLO-TRCA.

We propose a real-time detection model called YOLO-TRCA for abnormal crew behavior onboard based on the above analysis. This model improves the original YOLOv5s by incorporating TransformerBlock self-attention, CBAM attention mechanisms, and the CIoU loss function, thereby improving the accuracy of crew behavior recognition. The YOLO-TRCA structure is illustrated in Figure 10, consisting of the input, backbone, neck, and head. The input images consist of various data featuring different scenarios, including lighting variations, motion blur caused by image jitter, overlapping or occluded crew members, and complex backgrounds.

(1) The C3 module before SPPF in the backbone is replaced by the C3-TransformerBlock module. The input of backbone consists of images with a resolution of 640 × 640, including three RGB channels. The output features are four different scales: 128 × 80 × 80, 256 × 40 × 40, 384 × 20 × 20, and 512 × 10 × 10, which are fed into the neck.

(2) Three CBAMs are introduced at the input stage of the PAN in the neck. The input to CBAM1 is the 128 × 80 × 80 feature map output from the fourth layer C3 of the backbone, the input to CBAM2 is the 128 × 40 × 40 feature map output from the Conv module, and the input to CBAM3 is the 256 × 20 × 20 feature map output from the Conv module. The output feature maps from the three CBAM maintain the original scale.

(3) The inputs to the head are the four feature maps of sizes 128 × 80 × 80, 256 × 40 × 40, 384 × 20 × 20, and 512 × 10 × 10. The Detect output consists of tensors with dimensions 4 × 80 × 80 × 10, 4 × 40 × 40 × 10, 4 × 20 × 20 × 10, and 4 × 10 × 10 × 10. Here, 4 represents the number of anchors, and 10 indicates the values for 5 classes, confidence, and the xywh coordinates of the predicted boxes.

### 3.2. Video Sequence-Based Crew Identity Recognition

**Figure 11:**
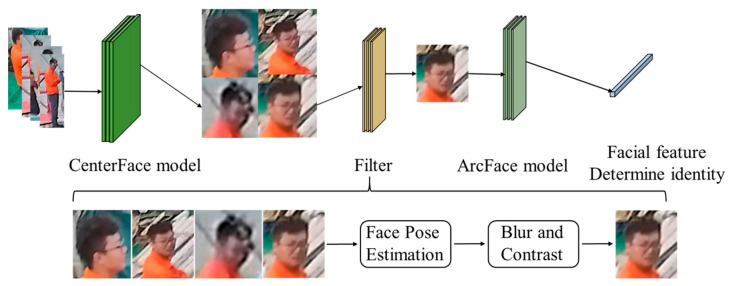
CFA: crew identity recognition process.

We need to determine the specific identity of the crew members through facial recognition once any abnormal behavior is detected. The environmental conditions on ships are intricate, and shipboard surveillance cameras are typically mounted in upper compartments and on monitoring poles. The distance between the camera deployment height and the personnel is more than 2.5 m, presenting a top-down shooting state. It is challenging to capture complete facial images of personnel in the videos, and the resolution of facial images is low. Common face recognition algorithms are not suitable for on-ship scenarios. We propose an identity recognition method (CFA) based on face quality assessment to improve the accuracy of crew identity recognition (Figure 11). The CenterFace model is utilized to detect faces in the video sequence, generating a sequence of facial images. A fast face quality assessment algorithm called Filter is designed to screen out high-quality facial images of personnel. Finally, Arcface is employed to achieve crew identity recognition.

#### 3.2.1. Filter: Fast Face Quality Assessment Algorithm

We design a fast face quality assessment algorithm called Filter in this paper, which performs real-time face quality assessment during video tracking. Filter calculates the face pose based on the relative positions of facial feature points of crew members in each frame of the video. It then selects face images with pose angles below a certain threshold. Subsequently, it computes the image blurriness and contrast to further filter out low-quality face images. As a result, high-quality image data are obtained for use in face recognition.

**Figure 12:**
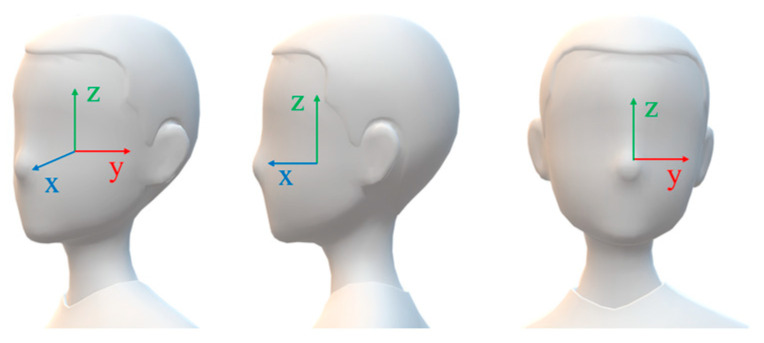
Facial coordinate diagram.

1. Fast Face Pose Estimation;

Face pose estimation uses the 2D image of crew members captured by a monocular camera to estimate the yaw and pitch angles of the face, which are two types of 3D information. As depicted in Figure 12, the 3D facial coordinate system P (x,y,z) has its origin centered at the head, with the Y-Z plane parallel to the camera imaging plane Π (y,z). The *X*-axis direction corresponds to the shooting direction. The yaw angle represents the rotation angle β of the face around the *Z*-axis. The pitch angle represents the rotation angle γ of the face around the *Y*-axis. The facial detection box is represented by the coordinates of two feature points: the top-left corner $f_{1}(x,y,z)$ and the bottom-right corner $f_{2}(x,y,z)$. The coordinates of the five facial feature points are the left eye $e_{1}(x,y,z)$, the right eye $e_{2}(x,y,z)$, the nasal tip $n_{1}(x,y,z)$, the left mouth corner $m_{1}(x,y,z)$, and the right mouth corner $m_{2}(x,y,z)$.

In the application of video surveillance on ships, the camera’s focal length typically ranges from 6 to 12 mm. Crew members are often situated at a considerable distance from the camera, typically exceeding 5 m. This results in a relatively small proportion of their faces being visible in the captured images. Based on imaging principles and the characteristics of crew members’ work behavior, the impact of varying imaging distances between frames on the proportion of facial images can generally be ignored. However, facial rotation can cause significant changes in the proportion of the face within the video images. Therefore, this algorithm focuses on considering the changes in the rotation angle of the face, while ignoring the changes in facial displacement.

**Figure 13:**
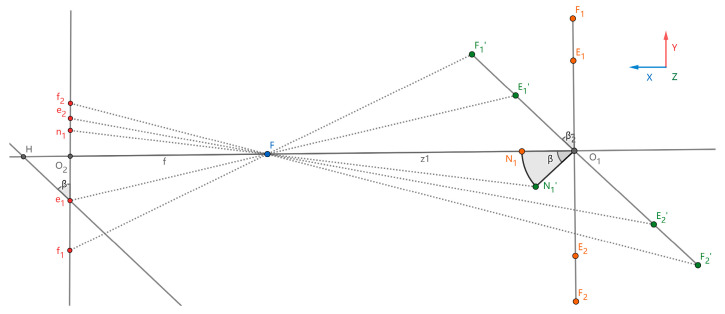
Yaw rotation.

The imaging process of a camera when a face rotates at a yaw angle is illustrated in Figure 13. During this process, the three-dimensional coordinates of various points are orthogonally projected onto the X–Y plane. Specifically, when the face is facing the camera directly, the facial spatial coordinates are orthogonally projected onto the X–Y plane, resulting in the coordinates $F_{1},F_{2},E_{1},E_{2},N_{1}$. After the face rotates at a yaw angle, the orthogonal projection coordinates become $F_{1}^{\prime },F_{2}\prime ,E_{1}^{\prime },E_{2}^{\prime },N_{1}\prime$, respectively, with the rotation angle being ∠β. Subsequently, after the camera imaging process, the *Y*-axis coordinates of these orthogonal projection points on the plane Π (y,z) are denoted as $f_{1},f_{2},e_{1},e_{2},n_{1}$, respectively.

As shown in Figure 13, let $O_{2}$ be the midpoint of $f_{1}f_{2}$. Draw a parallel line to $F_{1}߰F_{2}߰$ through $e_{1}$, intersecting the line $O_{1}O_{2}$ at point H. Then, $\cos\beta =e_{1}O_{2}/e_{1}H$. Through a face detection algorithm, the coordinates of five feature points on the imaging plane can be obtained. However, the length of $e_{1}H$ cannot be calculated when the angle β, the focal length f, and the image distance Z1 are unknown. The length of $e_{1}H$ varies with the change in β. Among the five facial points, $\max\;(f_{1}n_{1},f_{2}n_{1})$ and $\max\;(e_{1}n_{1},e_{2}n_{1})$ exhibit the same trend of variation, where max represents taking the maximum value. According to the literature [42], there is a relatively large error when using a convolutional neural network to locate the feature points $f_{1}f_{2}$. Therefore, the relationship between $\max\;(e_{1}n_{1},e_{2}n_{1})$ and $e_{1}H$ is analyzed as a priority. From Figure 13, it can be observed that $e_{1}O_{2}\leq \max\;(e_{1}n_{1},e_{2}n_{1})$. Let the error $\varepsilon =|\max\;(e_{1}n_{1},e_{2}n_{1})-e_{1}H|$, so $\underset{\beta \to 0}{\lim}\varepsilon \to 0$.

After simulating through a Python program, we obtained the following equation:

(2) $$\left|\frac{\varepsilon }{e_{1}H}|\leq 0.2,\;\beta \in (0,\frac{\pi }{3}\right)$$

where $e_{1}H$ represents the distance between points $e_{1}$ and H.

Therefore, when β is small, it is reasonable to approximate $e_{1}H$ using $\max\;(e_{1}n_{1},e_{2}n_{1})$. When β>π/3, the accuracy of face recognition is extremely low, and calculating the exact angle in this state is not significant. Based on the above analysis, the calculation formula for the β angle is given by the following Equation (3):

(3) $$\beta =arccos\;(\frac{0.5\times |e_{1}(y)-e_{2}(y)|}{\max\;(|e_{1}(y)-n_{1}(y)|,|e_{2}(y)-n_{1}(y)|)})\times \frac{180}{\pi },\beta \in (0,\frac{\pi }{3})$$

where $e_{1}(y)$, $e_{2}(y)$, and $n_{1}(y)$ represent the *Y*-axis coordinates of the left eye center, right eye center, and nose tip center in the two-dimensional image, respectively.

Referring to the pitch angle calculation process in reference [43], Equation (4) is as follows:

(4) $$\gamma =\arccos\;(\frac{\left|E_{1}E_{2}\right|}{\left|d\;(E_{1}E_{2},M)\right|})\times \frac{180}{\pi }$$

where $\left|E_{1}E_{2}\right|$ is the length of the line connecting the two eyes, M is the midpoint of the lips, and d is the distance from the point M to the line $\left|E_{1}E_{2}\right|$. Considering that the crew is far away from the camera in the application scenario, there are differences in the positions of the corresponding feature points extracted by the face detector compared with reference [43]. Moreover, the calculation accuracy of the pitch angle has a small impact on the accuracy of face recognition. To improve the calculation speed, we simplify the distance calculation process by replacing $\left|E_{1}E_{2}\right|$ with $\left|e_{2}(y)-e_{1}(y)\right|$ and $d\;(E_{1}E_{2},M)$ with $\left|e_{1}(z)-m_{1}(z)\right|$, as follows:

(5) $$\gamma =\arccos\;(\frac{\left|e_{2}(y)-e_{1}(y)\right|}{\left|e_{1}(z)-m_{1}(z)\right|})\times \frac{180}{\pi }$$

where $e_{1}(z)$, $e_{2}(z)$, and $n_{1}(z)$ represent the *Z*-axis coordinates of the left eye center, right eye center, and nose tip center in the two-dimensional image.

Following experimentation, we established the threshold ranges for the yaw and pitch angles of the face as follows:

(6) $$\left\{ \begin{array}{c} ❘\beta ❘<22.5{}^{\circ}\\ ❘\gamma ❘<30.5{}^{\circ} \end{array}\right.$$

2. Image Blur and Contrast Calculation;

We selected the Tenengrad function and contrast function to compute image quality for low-quality image filtering. The gradient function uses the Sobel operator to extract the gradient values in the horizontal and vertical directions, respectively.

(7) $$K_{x}=[\begin{array}{ccc} 0 & -1 & 0\\ 0 & 2 & 0\\ 0 & -1 & 0 \end{array}],K_{y}=[\begin{array}{ccc} 0 & 0 & 0\\ -1 & 2 & -1\\ 0 & 0 & 0 \end{array}]$$

Apply the Sobel operator for two convolutions on the image to obtain the blur index s.

(8) $$s=\sqrt{f^{2}(x,y)*K_{x}+f^{2}(x,y)*K_{y}}$$

In Equation (8), f (x,y) represents the grayscale value of the image at coordinates (x,y). Subsequently, the contrast of crew members’ facial images is calculated using the following contrast formula:

(9) $$c=\sum_{\delta }\delta {\left(i,j\right)}^{2}P_{\delta }(i,j)$$

where δ (i,j)=|i−j| represents the grayscale difference between adjacent pixels. And $P_{\delta }(i,j)$ denotes the probability distribution of pixels with a grayscale difference of δ between adjacent pixels.

The thresholds for blur measure s and contrast range c are determined through experiments as follows:

(10) $$\left\{ \begin{array}{c} s<\frac{32.5*\sum (x,y)}{15444}\\ 40<c<60 \end{array}\right.$$

where s represents the threshold for motion blur, c represents the threshold for contrast, and ∑(x,y) denotes the number of pixels in the input image. Here, 32.5 is a scaling factor used to adjust the calculation of the blur threshold. Furthermore, 15,444 is a normalized constant at 1080P resolution, used to convert the number of pixels into a scale suitable for clarity assessment.

#### 3.2.2. Crew Identity Recognition Method

**Figure 14:**
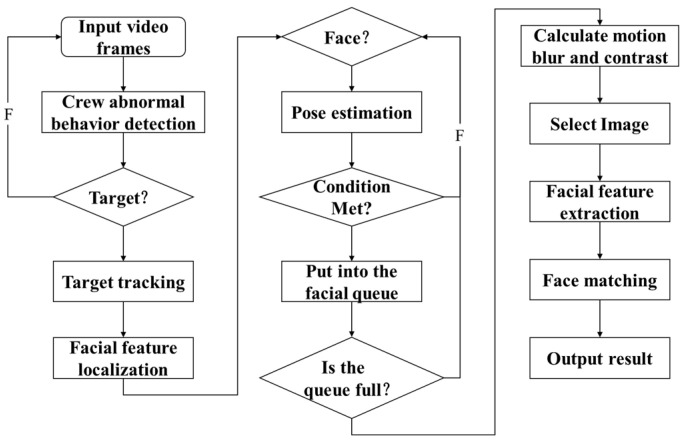
Diagram of the crew identity recognition process.

The Filter algorithm is used to screen the video, obtaining high-quality face image data for crew face recognition. The overall identification process is illustrated in Figure 14.

The pseudocode description of the crew identity recognition algorithm is shown in Algorithm 1.
**Algorithm 1** Crew Identity Recognition Algorithm for Video Sequences**Input**: $Image_{i}\in Video^{t_{1},t_{2},t_{3},\ldots ,t_{n}}$ **Output**: Abnormal behavior category of crew members C, crew number S, time T, image I, name NInitialize the crew facial feature database matrix M, its size is n×512
Initialize crew tracking mapping hash table H, including face image queue and crew ID**while** $Image_{i}$ has next **do**list = *CrewActionDetect*($Image_{i}$);//Detection for abnormal behavior of crew**for** each $list_{i}$ in list **do***isnew,id = DeepSORT*($Image_{i}$, $list_{i}$);//Deepsort for multi-target tracking**if** *isnew* **then***crewimage = CropImage*($Image_{i}$, $list_{i}$);//Crop crew image*Upload*(*crewimage,T,id*);//Upload crew image**end if****if** *H*[*id*].*new* **then**//No crew identity image has been uploaded*crewimage = CropImage*($Image_{i}$, $list_{i}$);*faceinfo = Centerface*(*crewimage*);//Centerface model for face detection**if** *faceinfo is not Empty* **then**$valid_{angle}$ = *AngleFilter*(*crewimage*, *faceinfo*);//Determine facial pose angle**if** $valid_{angle}$ **then***faceimage = CropImage*(*crewimage*,*faceinfo.loc*);//Crop facial image*H*[*id*].*add*(*faceimage*);//Add to the facial image queue**if** *H*[*id*].*size* > 5 **then**//Queue greater than 5 images*bestimage* = *QualityFilter*(*H*[*id*]);//Select images based on a contrast//close to 50 and in ascending order of blurriness*bestface* = *FaceAlignment*(*bestimage*,*faceinfo*)//Face alignment*f = Arcface*(*bestface*);//Arcface model extracts facial features*ans* = *Max*(*f*×*M*);//Calculate the confusion matrix,//select the maximum value
**if** *ans* > 0.65 **then***Upload*(*bestface,T,id*);//Upload crew identity image*H*[*id*].*new = False;***end if****end if****end if****end if****end if****end for****end while****return** None

## 4. Experimental Results and Analysis

### 4.1. Crew Abnormal Behavior Detection Experiment

#### 4.1.1. Experimental Dataset

**Figure 15:**
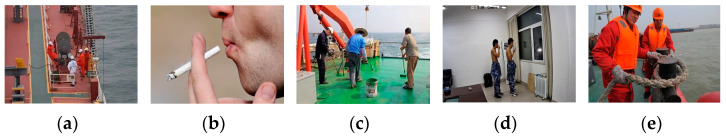
Partial images of the dataset: (**a**) not wearing a life jacket: nolifevast; (**b**) smoke; (**c**) not wearing work clothes: notrainlifevast; (**d**) not wearing a shirt: nocoat; (**e**) normal: lifevast.

The dataset was collected from real onboard surveillance images and some land-based scene images, covering complex scenarios on the ship, various camera angles, and different personnel postures. The dataset includes various scenes, such as those with significant lighting changes, scenes blurred due to image jitter, and overlapping and occluded crew scenarios, among others. It also includes behavioral data, such as wearing life jackets and smoking, extracted from the life jacket detection dataset [44] and smoking detection dataset [45] in PaddlePaddle AI Studio, respectively, enriching the custom dataset. The dataset is divided into training, testing, and validation sets with ratios of 60%, 20%, and 20%, respectively. Some images from the dataset are shown in Figure 15, with the targets categorized into five classes: not wearing a life jacket, smoking, not wearing work clothes, not wearing a shirt, and normal. Each image was annotated using the Labelimg tool. The training set consists of 3487 images from different scenarios, with specific proportions shown in Table 3, and over 8700 labeled targets (see Table 4).

#### 4.1.2. Comparative Experiments

The environmental parameters used in the experiment conducted in this paper are shown in Table 5.

The Mosaic data augmentation strategy was used to randomly alter training samples to improve model generalization. The batch size was set to 4. The training momentum was set to 0.937. The learning rate was set to 0.0001 with cosine annealing adjustment. The weight decay parameter was 0.005, and the number of training epochs was set to 150. Model performance was evaluated using mean average precision (mAP) and inference time to quantify accuracy and speed. The highest values during the training epochs were used. mAP@0.5:0.95 represents the average precision over the IoU threshold range [0.5, 0.95] with a step size of 0.5. mAP@0.5 represents the average precision when the IoU threshold is set to 0.5.

We conducted comparative experiments on different algorithm models to verify the performance of the crew abnormal behavior detection algorithm. The experimental results are shown in Table 6. The YOLO-TRCA algorithm achieved the highest accuracy, with mAP@0.5:0.95 and mAP@0.5 reaching 76.9% and 93.2%, respectively. It also had an inference time of 16.9 ms. YOLOv5s, AIA, CenterNet, and YOLOv4 did not exceed YOLO-TRCA in terms of accuracy. Only YOLOv5s had a lower inference time compared to YOLO-TRCA, while AIA, CenterNet, and YOLOv4 had longer inference times. This indicates that YOLO-TRCA achieves a good balance between high accuracy and a low inference time.

**Figure 16:**
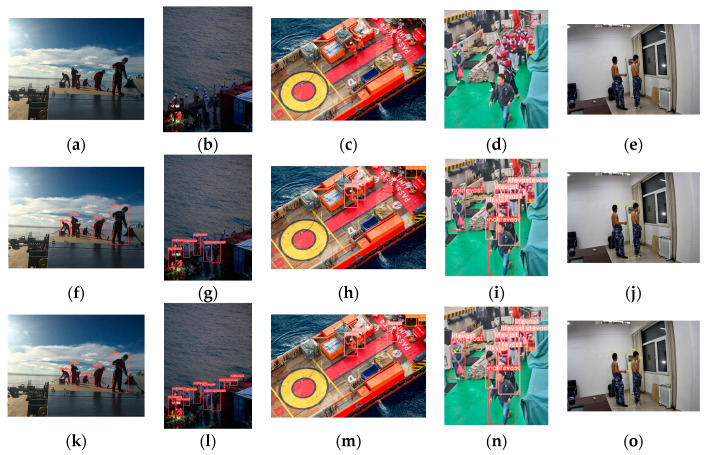
Comparison of detection results: (**a**) original image; (**b**) original image; (**c**) original image; (**d**) original image; (**e**) original image; (**f**) YOLOv5s; (**g**) YOLOv5s; (**h**) YOLOv5s; (**i**) YOLOv5s; (**j**) YOLOv5s; (**k**) proposed method; (**l**) proposed method; (**m**) proposed method; (**n**) proposed method; (**o**) proposed method.

A comprehensive analysis of Figure 16 and Table 7 shows that, under conditions of varying onboard lighting interference, image jitter, complex backgrounds, and crew occlusions with small proportions, the proposed method improves detection accuracy by an average of 35.0% compared to YOLOv5s. In the detection comparison experiment shown in Figure 16a, the proposed method successfully identified the furthest crew member not wearing a life jacket, but the occluded crew member was not accurately distinguished. When considering Figure 16b, the method detected all 11 crew members without life jackets, even under conditions of local overexposure, underexposure, or blur. The complex scene presented in Figure 16c saw the proposed method effectively detecting two crew members in the upper right corner who were not wearing work clothes. In the scenario depicted in Figure 16d, where crew members were significantly overlapping, the method detected three more normal crew members compared to YOLOv5s. However, one crew member without a life jacket was misidentified as normal, while another normal crew member was incorrectly labeled as not wearing a life jacket. Figure 16e illustrates that the proposed method successfully detected two crew members who were shirtless and smoking. In conclusion, the method proposed in this paper shows enhancements over previous methods in detecting abnormal behavior of crew members. However, it still faces challenges, including missed detections of obstructed crew members and false positives in determining whether crew members are wearing life jackets. To enhance the differentiation ability between overlapping crew members and those with similar features, further improvements in the network’s feature extraction capability can be implemented. This could involve optimizing the architecture, incorporating advanced techniques, like attention mechanisms, or leveraging additional training data to capture more nuanced characteristics of the crew members.

#### 4.1.3. Ablation Experiment

Ablation experiments were conducted to validate the effectiveness of the improved model structure, using mAP@0.5:0.95 as the accuracy metric and inference time as the speed metric. As shown in Table 8, the model with C3-TransformerBlock + 3 CBAM + CIoU-NMS achieved the best accuracy, with a 4.2% increase in precision and an additional 0.7 ms in inference time. With only CIoU-NMS, the model achieved a 0.5% increase in accuracy and an additional 0.4 ms in inference time, suggesting improved performance in occlusion scenarios. The model with the C3-TransformerBlock had a 2.4% increase in accuracy and an additional 0.1 ms in inference time, demonstrating that the C3-TransformerBlock enhances feature extraction capability in the backbone network. Adding 3 CBAM led to a 1.3% accuracy improvement and an extra 0.2 ms in inference time, indicating that CBAM enhances focus on crew regions. Overall, within the acceptable range of increased model inference time, the model’s accuracy significantly improved, proving the effectiveness of the YOLO-TRCA structural modifications.

**Figure 17:**
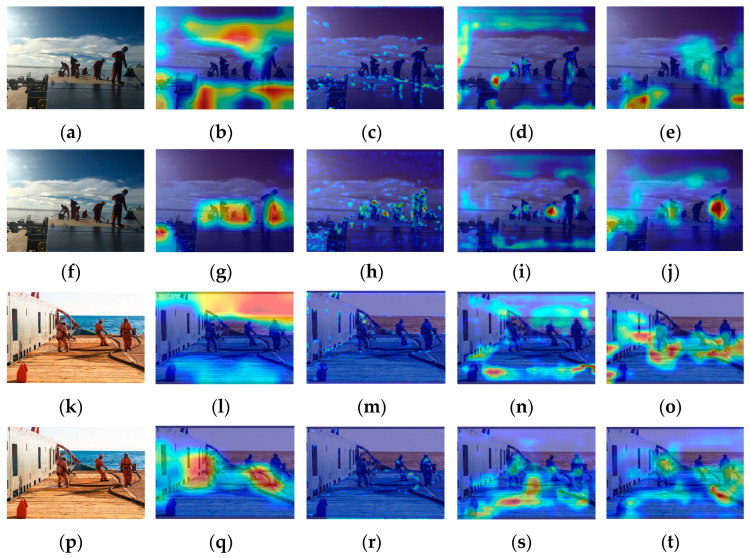
Features visualization of the network: (**a**) original image; (**b**) C3; (**c**) before adding CBAM1; (**d**) before adding CBAM2; (**e**) before adding CBAM3; (**f**) original image; (**g**) C3-TransformerBlock; (**h**) after adding CBAM1; (**i**) after adding CBAM2; (**j**) after adding CBAM3; (**k**) original image; (**l**) C3; (**m**) before adding CBAM1; (**n**) before adding CBAM2; (**o**) before adding CBAM3; (**p**) original image; (**q**) C3-TransformerBlock; (**r**) after adding CBAM1; (**s**) after adding CBAM2; (**t**) after adding CBAM3.

The difference in feature visualization between the original C3 module and the improved C3-TransformerBlock module is significant. Figure 17b,l shows that the self-attention generated by the C3 module is entirely focused on environmental factors. In contrast, the C3-TransformerBlock in Figure 17g,q distributes attention to both environmental factors and the entire crew area. Therefore, the addition of the C3-TransformerBlock enhances the feature extraction capability of the YOLOv5 backbone network and provides a clear attention guidance for target features. Figure 17 shows the visualization results before and after the addition of CBAM. Prior to adding CBAM, the attention towards the crew members was low, and background features severely interfered with the target features. The attention towards the crew members significantly increased after incorporating CBAM. The above phenomenon indicates that introducing three CBAM modules into the PAN input of the feature fusion network effectively suppresses environmental noise and enhances crew feature representation, thereby improving the model’s detection accuracy.

### 4.2. Comparative Experiment on Crew Identity Recognition

The data for the identity recognition experiment were collected from real crew face videos on the ship, with a recognition threshold set at 0.65. This section conducts comparative experiments on crew face recognition using the face detectors Centerface and MTCNN, the face feature extraction models Arcface and VGG-Face, the pose estimation algorithm POSIT, and the face quality evaluation algorithm (Filter) designed in this paper. In the experiment, the input face images to the face detector were all scaled to 112 × 112. After detecting the faces, facial landmarks were used for alignment, and the aligned faces were then fed into the recognition model for feature extraction and identification. The evaluation metrics for the algorithm include true match rate (TMR), false match rate (FMR), and false rejection rate (FRR). These metrics quantify the accuracy of the algorithm, while the inference speed measures its processing speed.

Table 9 shows that the algorithm with the highest accuracy is TL-GAN + VGG-Face, achieving a TMR of 74%, but with an FMR and FRR of 12% and 14%, respectively. The algorithm with the lowest accuracy is MTCNN + FaceRecognition, with TMR, FMR, and FRR values of 19%, 8%, and 73%, respectively. The fastest algorithm is CenterFace + Arcface, with a processing time of 15.82 ms, while the slowest is TL-GAN + VGG-Face, taking 15,100 ms. The method used in this paper is CenterFace + Filter + Arcface, achieving a TMR of 68%, an FMR of 24%, and an FRR of 8%. Compared to CenterFace + Arcface, accuracy is significantly improved with an additional processing time of 0.54 ms. While the accuracy shows a slight decrease relative to TL-GAN + VGG-Face, there is an improvement in speed by 15,083.65 ms, or 923.54 times. Both accuracy and speed see significant enhancements compared to MTCNN + FaceRecognition and MTCNN + POSIT + FaceRecognition. The above analysis demonstrates the effectiveness of the Filter algorithm.

The results indicate that the method proposed in this paper achieves a good balance between real-time performance and algorithm accuracy, making it suitable for real-time video crew face recognition scenarios.

### 4.3. Actual Testing on Board

**Figure 18:**
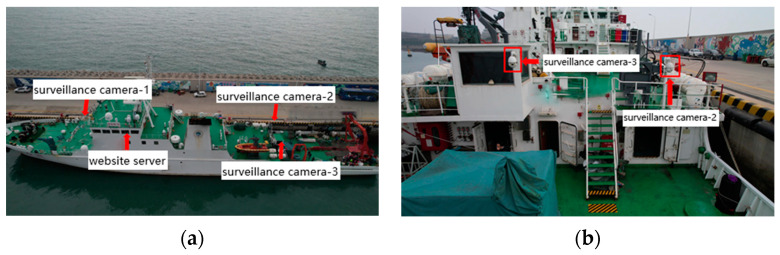
Marine equipment layout diagram: (**a**) overall picture; (**b**) partial view.

An abnormal crew monitoring and analysis system was designed to meet the needs of onboard managers in practical applications. This system enables the monitoring, capturing, statistical analysis, and visualization of a crew’s abnormal behaviors. After the system design and development were completed, both the software and hardware of the system were deployed and installed on test vessels, connected to the vessel’s wired local area network. To effectively monitor the crew on the fore and aft decks of the test vessels, the deployment scheme included one onboard camera for the foredeck and two onboard cameras for the aft deck, with the web server placed in the upper bridge. The specific deployment layout is shown in Figure 18.

**Figure 19:**
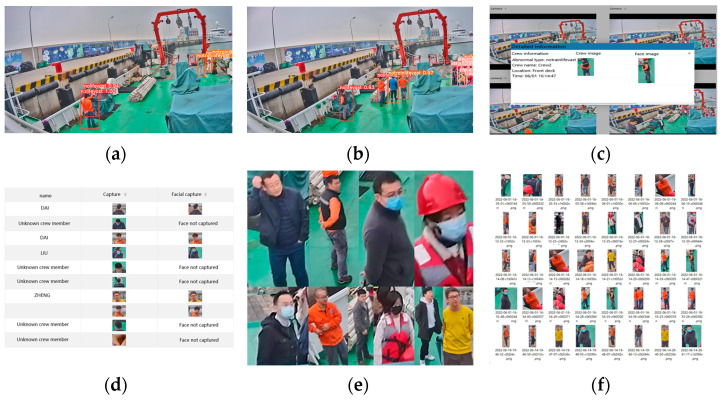
Algorithm effect and software design: (**a**) abnormal behavior detection of crew members in monitoring; (**b**) abnormal behavior detection of crew members in monitoring; (**c**) capturing and identifying abnormal crew; (**d**) abnormal crew identification record; (**e**) abnormal crew identity recognition results; (**f**) abnormal crew identity recognition results.

The resolution of the onboard monitoring video was set to 1920 × 1080. The experimental period was from 17:00 on February 4th to 08:00 on February 13th, with a duration of 183 h. Table 10 shows that during the testing period, a total of 483 images of abnormal crew behavior and 336 images with correct identity recognition were captured, resulting in an identity recognition accuracy of 69.6% for abnormal crew members. The accuracy of crew abnormal behavior recognition is presented in Table 11, with an average accuracy of 92.3%. The system takes an average of 39.5 ms to process one frame, and the website response delay is controlled within a range from 0.68 ms to 3.11 ms. Figure 19a,b demonstrates the effectiveness of detecting abnormal behavior among crew members. Figure 19c,d shows the software interface for identifying abnormal crew members, and the identification results are presented in Figure 19e,f. Based on the test results, the abnormal crew monitoring and analysis system is fully capable of real-time detection of a crew’s abnormal behaviors and recognition of crew identity.

## 5. Conclusions

This paper proposes an abnormal crew detection network (ACD-Net) for real-time detection and recognition of abnormal crew members in realistic complex ship scenes. The ACD-Net is tested for identifying abnormal crew members on actual ships to verify its effectiveness. The experimental results demonstrate that ACD-Net achieves an abnormal behavior detection accuracy of 92.3% and an identity recognition correct matching rate of 69.6%. At a 1080P resolution, the per-frame processing time is no more than 39.5 ms, providing high accuracy and lower processing time compared to other methods.

The ACD-Net proposed in this paper also has certain limitations. The application ratio in real ship environments needs further improvement to validate the algorithm’s generalization ability in various scenarios, such as fog and low light. The types of abnormal behavior recognized are still relatively few, and further collection of additional abnormal behaviors is necessary to assess recognition performance. Additionally, the accuracy of detecting abnormal behavior and identity recognition for obstructed crew members still requires enhancement.

## Figures and Tables

**Table 1:** Performance evaluation on different datasets.

Dataset	YOLOv5	Faster R-CNN	SSD	RetinaNet	YOLOv4
COCO	56.8%	57.5%	51.5%	54.2%	43.5%
Pascal VOC	82.1%	80.9%	77.6%	80.5%	85.1%
ImageNet	74.2%	73.9%	71.8%	72.5%	69.2%

**Table 2:** Real-time inference speed comparison.

Model	YOLOv5	Faster R-CNN	SSD	RetinaNet	YOLOv4
Model input	640 × 640	1000 × 600	300 × 300	800 × 800	608 × 608
NVIDIA GeForce GTX 1080 Ti	60 FPS	3 FPS	25 FPS	7 FPS	43 FPS

**Table 3:** The proportion of different scenarios in the training set.

Different Scenarios	Brightness Variation Scene	Blurred Scenes with Image Jitter	Obstructive and Overlapping Scenes	Other Scenes
Number	1011	209	1186	1081
Proportion	29%	6%	34%	31%

**Table 4:** Number of classes in the training set.

Class	Abnormal				Normal
	Nolifevast	Notrainlifevast	Smoke	Nocoat	Lifevast
Target number	4200	1400	1000	600	1500

**Table 5:** Experimental machine configuration and compilation environment.

Experimental Hardware Configuration	Experimental Software Configuration
CPU: Intel(R) Core(TM) i7-10750H CPU @ 2.60 GHz GPU: Nvidia RTX 2060 6 G Laptop Memory: 16 GB DDR4 2933 MHz Hard Disk: 1 TB SSD	Python 3.6.12 CUDA 11.5 CUDNN 8.6.5 Pytorch 1.11.0 Tensorflow 1.13.0

**Table 6:** Comparison of detection methods for abnormal behavior of crew members.

Model	mAP@0.5:0.95/%	mAP@0.5/%	Inference Time/ms
YOLO-TRCA	76.9	93.2	16.90
YOLOv5s	72.7	91.8	16.20
AIA	74.4	92.8	129.03
CenterNet	71.2	92.5	42.00
YOLOv4	70.3	88.7	26.31

**Table 7:** Statistics of abnormal behavior detection results for crew members.

	Image	Figure 16a	Figure 16b	Figure 16c	Figure 16d	Figure 16e
Class						
nolifevast	YOLOv5s (error)	4 (0)	6 (0)	—	2 (1)	—
	YOLO-TRCA (error)	5 (0)	11 (0)	—	2 (1)	—
	Targets	6	11	—	2	—
notrainlifevast	YOLOv5s (error)	—	—	1 (0)	—	—
	YOLO-TRCA (error)	—	—	2 (0)	—	—
	Targets	—	—	2	—	—
smoke	YOLOv5s (error)	—	—	—	—	0 (0)
	YOLO-TRCA (error)	—	—	—	—	2 (0)
	Targets	—	—	—	—	2
nocoat	YOLOv5s (error)	—	—	—	—	2 (0)
	YOLO-TRCA (error)	—	—	—	—	2 (0)
	Targets	—	—	—	—	2
lifevast	YOLOv5s (error)	—	—	2 (0)	5 (1)	—
	YOLO-TRCA (error)	—	—	3 (0)	8 (1)	—
	Targets	—	—	3	11	—
Precision improvement		16.7%	45.5%	40.0%	23.0%	50.0%

**Table 8:** Ablation experiments.

C3-TransformerBlock	CBAM-1	CBAM-2	CBAM-3	CIoU-NMS	mAP@0.5:0.95/%	Inference Time/ms
×	×	×	×	×	72.7—	16.2—
×	×	×	×	√	73.2↑	16.6↑
√	×	×	×	√	75.6↑	16.7↑
√	√	√	√	√	76.9↑	16.9↑

**Table 9:** Identity recognition performance.

Method	TMR	FMR	FRR	Inference Time/ms
CenterFace + Filter + Arcface	0.68	0.24	0.08	16.35
CenterFace+ Arcface	0.40	0.56	0.04	15.82
MTCNN + FaceRecognition	0.19	0.08	0.73	432.18
MTCNN + POSIT + FaceRecognition	0.65	0.23	0.12	438.59
TL-GAN + VGG-Face	0.74	0.12	0.14	15,100

**Table 10:** Marine application experiment.

Test Project	Test Result
Number of abnormal crew image captures	483 times
Abnormal crew identity recognition frequency	336 times
Average frame time for video processing	39.5 ms
Website response delay	0.68 ms~3.11 ms

**Table 11:** Accuracy of detecting abnormal behavior of crew members.

Class	Abnormal				Normal
	Nolifevast	Notrainlifevast	Smoke	Nocoat	Lifevast
Accuracy/%	0.987	0.988	0.661	0.995	0.985

## Data Availability

The original contributions presented in this study are included in the article; further inquiries can be directed to the corresponding authors.

## Appendix A

**Table A1:** CBAM parameter selection experiment.

**(r,k)**	**P**	**R**	**mAP@0.5**	**mAP@0.5:0.95**
(32,7)	94.6%	92.0%	92.5%	76.6%
(16,7)	95.6%	91.7%	93.2%	76.9%
(8,7)	94.2%	91.5%	92.2%	76.7%
(32,3)	94.5%	91.2%	92.4%	76.6%
(16,3)	95.3%	91.5%	92.6%	76.6%
(8,3)	95.2%	91.8%	92.9%	76.2%

The size of the spatial convolution kernel is k, and the channel dimension reduction coefficient is r.
